# Risk factors for ICU-acquired weakness in sepsis patients: A retrospective study of 264 patients

**DOI:** 10.1016/j.heliyon.2024.e32253

**Published:** 2024-05-31

**Authors:** Jiajiao Liu, Zhaoxia Xu, Shuhong Luo, Yujie Bai, Jian Feng, Fuxiang Li

**Affiliations:** a Department of Critical Care Medicine, The General Hospital of Western Theater Command PLA, Chengdu, 610036, China; b Department of Emergency Department, The General Hospital of Western Theater Command PLA, Chengdu, 610036, China

**Keywords:** Sepsis, Intensive care unit, Acquired weakness, Risk factors, Diagnostic threshold

## Abstract

**Background:**

Sepsis is a common critical illness in intensive care unit (ICU) and an important risk factor for intensive care unit-acquired weakness (ICU-AW). The objective of the study is to analyze the risk factors of ICU-AW in septic patients.

**Methods:**

A total of 264 septic patients admitted to the General Hospital of the Western Theater Command from January 2018 to April 2022 were included in this study. The cohort was divided into 2 groups according to the presence or absence of ICU-AW. Clinical characteristics included age, sex, body mass index, length of ICU stay, multiple organ dysfunction syndrome, acute physiology and chronic health evaluation Ⅱ (APACHE Ⅱ), mechanical ventilation time, intubation, tracheotomy, protective constraint, lactic acid, fasting blood glucose, etc. The clinical characteristics of sepsis were evaluated using logistic regression analysis.

**Results:**

A total of 114 septic patients suffered ICU-AW during their ICU stay. Multivariate binary logistic regression analysis showed that APACHE Ⅱ score, mechanical ventilation time, protective constraint, and lactic acid were independent risk factors for ICU-AW in septic patients. The areas under the receiver operating characteristic curve (AUCs) were 0.791, 0.740 and 0.812, all P < 0.05, and the optimal cut-off values were 24 points, 5 days and 2.12 mmol/L, respectively.

**Conclusions:**

A high APACHE Ⅱ score, long mechanical ventilation time, protective constraint and high lactate concentration are independent risk factors for ICU-AW in septic patients. An APACHE Ⅱ score greater than 24 points, mechanical ventilation time longer than 5 days and lactate concentration higher than 2.12 mmol/L are likely to cause ICU-AW.

## 1 Background

Intensive care unit-acquired weakness (ICU-AW) is an unexplained widespread limb weakness syndrome that occurs in patients during the intensive care unit, which has no plausible cause other than the critical illness and relevant treatments [1]. ICU-AW is a common complication of critical illness, and the prevalence in the ICU ward is approximately 25–31 %, resulting in prolonged hospitalization and increased mortality [2]. It is different from various primary neuromuscular diseases, such as Guillain‒Barré Syndrome, amyotrophic lateral sclerosis, myasthenia gravis or multiple sclerosis. Muscle weakness or even atrophy develops as a secondary disorder [3]. ICU-AW also known as severe polyneuromyopathy, when nerve involvement is predominant, ICU-AW is sometimes called severe neuromyopathy (CIP); when muscle involvement is severe, ICU-AW is sometimes called myopathy gravis (CIM) [4]. In recent years, this invisible but far-reaching disease has received increasing attention. Many factors are considered to have a certain correlation with the occurrence of ICU-AW, including sepsis, prolonged mechanical ventilation, hyperglycemia, neuromuscular blockers, and the use of vasoactive drugs [[5], [6], [7], [8], [9], [10]]. Sepsis defined as critical organ dysfunction caused by a dysregulated host response to infection, is a leading cause of ICU admission. Sepsis patients are the main population in ICU. In recent years, an increasing number of septic patients have survived after treatment but new functional disabilities in activities of daily living can persist for a long time after discharge [11]. Compared with other patients, the sepsis population has a higher proportion of adverse events for developing muscle weakness. Some progress has been made in the research on risk factors for ICU-AW in mechanically ventilated patients. Neuromuscular blocking agents and Sedatives such as propofol have also been shown to increase the incidence of ICU-AW in mechanically ventilated patients [[12], [13], [14]]. However, there is still a lack of research concerning ICUAW in septic patients. The current pathogenesis of ICU-AW is not clear, and clinical specific indicators are also insufficient. The traditional electrophysiological examination is relatively complicated, time-consuming and professional-required. It is not easy to promote it in many conditions. Therefore, it is particularly important to explore risk factors to prevent the occurrence of ICU-AW in sepsis patients. Patients often suffer reduced muscle strength and impaired exercise tolerance during their ward stay or after discharge, but the possible risk factors for ICU-AW among septic patients have been explored few.

This study retrospectively analyzed the case data of sepsis patients in the ICU of our hospital to identify the risk factors for ICU-acquired weakness in sepsis patients. Each independent risk factor was evaluated by the receiver operating characteristic curve (ROC curve) to provide a clinical reference.

## 2 Methods

The six muscle groups of the body (bilateral wrist extension, forearm flexion, shoulder abduction, foot dorsiflexion, knee extension, thigh flexion) were graded, and the muscle strength of each group of the body was measured according to the Oxford muscle strength scale, with a total score of 60 points. Muscle strength assessment is performed on the day before and the day of discharge or transferring to the general ward. When both Medical Research Council sum score (MRC-SS) was less than 48 and the interval between the two scores was 24 h, the patient was diagnosed with ICU-AW [15]. The assessment method requires the patient to have a sufficient level of consciousness to cooperate and respond to orders. In this study, the MRC-SS was determined for the enrolled patients through exclusion and inclusion criteria. Sepsis patients who met the diagnosis of ICU-AW during ICU hospitalization were classified into the ICU-AW group, and patients who did not meet the diagnosis criteria were classified into the non-ICU-AW group. This study was conducted in accordance with the Declaration of Helsinki (as revised in 2013) and was approved by the Institutional Ethics Board of Western Theater General Hospital (2022EC2-ky035). All participants provided written informed consent to participate in this study.

### 2.1 Patient selection

#### 2.1.1 Inclusion criteria

Patients were selected if they met the following inclusion criteria: (Ⅰ) met the Sepsis 3.0 diagnostic criteria; and (Ⅱ) were over 18 years of age.

#### 2.1.2 Exclusion criteria

Patients were excluded if they presented with the following: (Ⅰ) patients who did not meet the above diagnostic criteria; (Ⅱ) ICU ward stay less than 48 h; (Ⅲ) patients who had craniocerebral trauma, spinal cord injury, cerebrovascular disease, brain tumor, Guillain‒Barré Syndrome, multiple limb fractures or open wounds, central nervous system infection and other diseases that can cause neuromuscular incapacity.

**Fig. 1:**
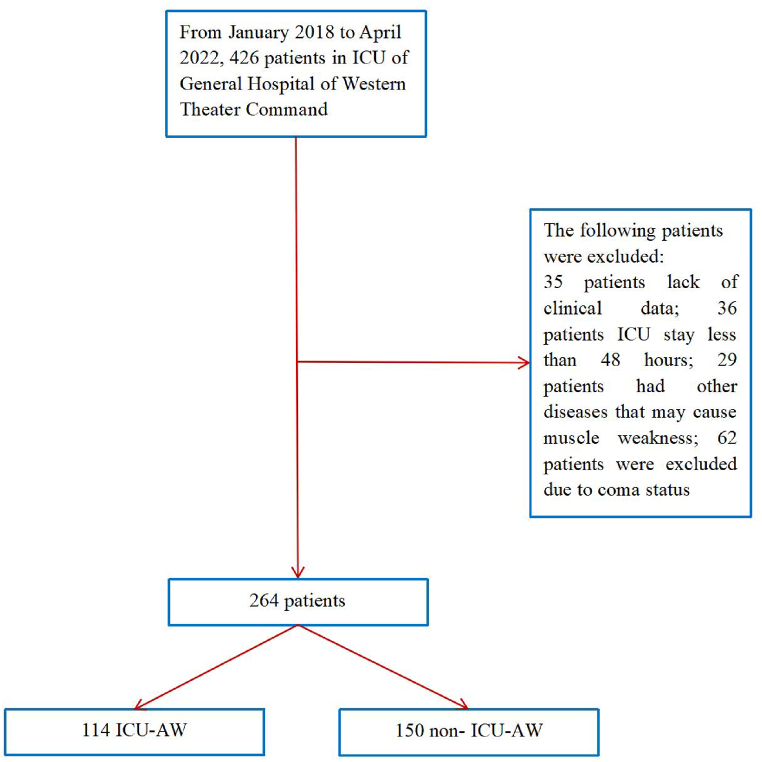
Flowchart detailing enrollment of subjects.

Study enrollment is outlined in Fig. 1. Of the 426 patients from January 2018 to April 2022 who met the inclusion criteria, 35 patients were excluded because of missing clinical data. Of the remaining patients, 36 ICU wards stay shorter than 48 h. Another 29 patients were excluded because of neurological disorders that might contributed to muscle weakness on admission. Another 62 patients were excluded owing to disturbance of consciousness, which could not be diagnosed as ICU-AW by MRC-SS. The remaining 264 patients met the final inclusion criteria.

### 2.2 Measures and statistics

#### 2.2.1 Measures

Patients who were included in our final analysis were divided into two groups according to the presence of ICU-AW. The following possible predictors for the occurrence of ICU-AW in septic patients were assessed based on previous studies and experiences: patients' general conditions, the therapy they received and laboratory examination indexes. All predictive factors that may lead to the occurrence of ICU-AW in patients with sepsis were collected until the patients were discharged or transferred to the general ward. Laboratory examination indexes were assessed by recording the daily results during this period and calculating their average value. The patient's general conditions included age, sex, BMI, acute physiology and chronic health evaluation Ⅱ (APACHE Ⅱ), length of ICU stay, comorbidities (such as diabetes, DIC and septic shock), and multiple organ dysfunction syndrome (MODS). The therapy they received included protective constraints, mechanical ventilation time, continuous renal replacement therapy (CRRT), intubation, tracheotomy, ventilation, vasoactive agents, glucocorticoids, sedatives or analgesics, and muscle relaxants. Laboratory examination indexes included lactic acid, fasting blood glucose, blood calcium, procalcitonin (PCT) and serum albumin. Univariate regression analysis was performed on general conditions and laboratory examination indexes in the two groups of patients, followed by multivariate analysis for P ≤ 0.1 to identify the possible risk factors for ICU-AW among septic patients. Statistically significant continuous variables were converted to dichotomous variables using ROC curves. The appropriate cut-off points of each item were determined by ROC curves corresponding to the curve nearest the graph's upper left corner.

#### 2.2.2 Statistical analysis

Data were analyzed by SPSS version 25.0 software. Independent sample T tests were used to compare continuous variables, and χ^2^ tests were used where appropriate to compare categorical characteristics. Assessment of the difference in the main confection sites between the two groups, with analysis using χ^2^. To assess each clinical characteristic's absence on the occurrence of ICU-AW, logistic regression analysis was conducted, correcting for risk factors that showed a significant trend (P ≤ 0.1) in univariate analysis. The Hosmer‒Lemeshow test was performed to assess the goodness of fit. The ROC curve analysis determined the threshold values and the appropriate cut-off points for continuous variables. Calculating the sensitivity and specificity for each factor was followed to evaluate the prevalence of the included characteristics.

## 3 Results

### 3.1 Patient population

A total of 114 patients presented ICU-AW at ICU discharge, including 80 males and 34 females. The remaining 150 patients, including 100 males and 50 females, did not develop ICU-AW. The baseline characteristics of all patients in the ICU-AW and non-ICU-AW groups were comparable (see Table 1). The main infection sites of the two groups showed no significant difference (see Table 2).

**Table 1:** Comparison of baseline characteristics between patients with and without ICU-AW.

Characteristics	ICU-AW (n = 114, 43.2 %)	non-ICU-AW(n = 150, 56.8 %)	P-value
Age (year)	59.37 ± 12.37	59.15 ± 15.49	0.903
Sex (n, %)			0.544
Male	80 (70.2 %)	100 (66.7 %)	
Female	34 (29.9 %)	50 (33.3 %)	
BMI (kg/㎡)	23.33 ± 3.38	23.17 ± 3.89	0.741
APACHE Ⅱ	27.19 ± 5.99	20.19 ± 6.93	<0.001*
Length of ICU stay (day)	14.48 ± 11.48	7.67 ± 7.79	<0.001*
Comorbidities (n, %)			
Diabetes	43 (37.7 %)	44 (29.3 %)	0.151
DIC	47 (41.2 %)	49 (32.7 %)	0.152
Septic shock	79 (69.3 %)	84 (56.0 %)	0.028*
MODS (n, %)	82 (71.9 %)	99 (66.0 %)	0.304
Protective constraint (n, %)	78 (68.4 %)	40 (26.7 %)	<0.001*
Mechanical ventilation time (day)	8.18 ± 3.91	4.63 ± 5.00	<0.001*
CRRT (n, %)	53 (46.5 %)	57 (38.0 %)	0.166
Intubation (n, %)	80 (70.2 %)	92 (61.3 %)	0.135
Tracheotomy (n, %)	39 (34.2 %)	40 (26.7 %)	0.185
Vasoactive agent (n, %)	96 (84.2 %)	115 (76.7 %)	0.130
Glucocorticoid (n, %)	90 (78.9 %)	98 (65.3 %)	0.016*
Sedative/Analgesic (n, %)	90 (78.9 %)	108 (72.0 %)	0.197
Muscle relaxant (n, %)	54 (47.4 %)	51 (34.0 %)	0.028*
Lactic acid (mmol/L)	4.08 ± 2.06	2.40 ± 2.23	<0.001*
Fasting blood glucose (mmol/L)	9.38 ± 2.61	8.87 ± 2.44	0.105
Blood calcium (mmol/L)	2.00 ± 0.16	1.98 ± 0.25	0.547
CRP (mg/L)	148.67 ± 78.59	144.20 ± 88.09	0.669
PCT (ng/L)	34.38 ± 51.15	25.93 ± 32.21	0.102
BNP (pg/ml)	666.71 ± 829.21	552.26 ± 654.54	0.211
Serum albumin (g/L)	29.48 ± 3.78	28.82 ± 4.20	0.186

*, P < 0.05. BMI, body mass index; APACHE Ⅱ, acute physiology and chronic health evaluation Ⅱ; DIC, disseminated intravascular coagulation; MODS, multiple organ dysfunction syndrome; CRRT, continuous renal replacement therapy; CRP, C-reactive protein; PCT, procalcitonin; BNP, brain natriuretic peptide.

**Table 2:** Comparison of Main affection sites between patients with and without ICU-AW.

Main site of infections	ICU-AW	non-ICU-AW	P-value
Lung	72(63.2 %)	101(67.3 %)	0.244
Enterocoelia	12(10.5 %)	13(8.7 %)	
Gastrointestinal tract	8(7.0 %)	9(6.0 %)	
Biliary tract	7(6.1 %)	7(4.7 %)	
Blood	5(4.4 %)	7(4.7 %)	
Urinary system	4(3.5 %)	5(3.3 %)	
Skin soft tissue	6(5.3 %)	8(5.3 %)	

P < 0.05 has statistical significance. Chi-square test.

### 3.2 Results of univariate and multivariate logistic regression analysis

**Fig. 2:**
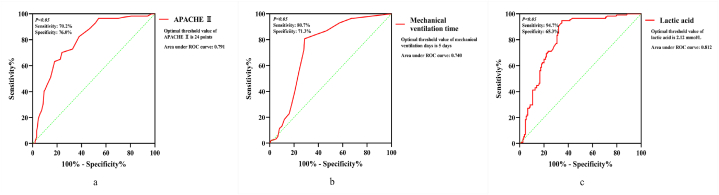
ROC curve of APACHE Ⅱ score, mechanical ventilation time and lactic acid. (a) APACHE Ⅱ. (b) Mechanical ventilation time. (c) Lactic acid.

Univariate analysis found that septic patients with ICU-AW had a higher APACHE Ⅱ score, longer mechanical ventilation time, higher concentration of lactic acid and higher incidence of septic shock (see Table 3). Additionally, the use of glucocorticoid and muscle relaxant medicines differed between the two groups. More septic patients with ICU-AW received protective constraints during their ICU admission compared with those who did not present ICU-AW. Other characteristics were not different between the two groups. Based on the univariate analysis, multivariate analysis revealed that APACHE Ⅱ score, mechanical ventilation time, protective constraint and lactic acid were independent risk factors for the occurrence of ICU-AW in septic patients (see Table 4). ROC curves showed that the diagnostic threshold of APACHE Ⅱ score was 24 points (sensitivity, 0.702; specificity, 0.760), mechanical ventilation time was 5 days (sensitivity, 0.807; specificity, 0.713), and lactic acid was 2.12 mmol/L (sensitivity, 0.947; specificity, 0.653) (see Fig. 2[a–c]).

**Table 3:** Univariate binary logistic regression analysis of risk factors between patients with and without ICU-AW.

Characteristics	Crude odds ratio (OR)	95 % CI	P value
Age	1.001	0.984–1.018	0.903
Sex	0.850	0.503–1.438	0.544
BMI	1.011	0.946–1.081	0.740
APACHE Ⅱ	1.172	1.120–1.226	<0.001*
Length of ICU stay	1.103	1.059–1.149	<0.001*
Mechanical ventilation time	1.179	1.112–1.250	<0.001*
Diabetes	1.459	0.870–2.446	0.152
Septic shock	1.773	1.063–2.960	0.028*
DIC	1.446	0.872–2.397	0.153
MODS	1.320	0.777–2.243	0.305
Protective constraint	5.958	3.487–10.180	<0.001*
Intubation	1.486	0.883–2.492	0.136
Tracheotomy	1.430	0.842–2.429	0.186
CRRT	1.418	0.865–2.324	0.166
Glucocorticoid	1.990	1.134–3.490	0.016*
Muscle relaxant	1.747	1.060–2.878	0.029*
Lactic acid	1.487	1.283–1.724	<0.001*
Fasting blood glucose	1.084	0.983–1.195	0.106
Blood calcium	1.421	0.454–4.449	0.546
PCT	1.005	0.999–1.011	0.107
Serum albumin	0.959	0.901–1.020	0.186

*, P < 0.1. BMI, body mass index; APACHE Ⅱ, acute physiology and chronic health evaluation Ⅱ; DIC, disseminated intravascular coagulation; MODS, multiple organ dysfunction syndrome; CRRT, continuous renal replacement therapy; PCT, procalcitonin; OR, odds ratio.

**Table 4:** Multivariate binary logistic regression analysis of risk factors between patients with and without ICU-AW.

Characteristics	Adjusted odds ratio (OR)	Regression coefficients (β)	95 % CI	P value
APACHE Ⅱ score	1.116	0.110	1.063–1.172	<0.001*
Length of ICU stay	1.040	0.039	0.988–1.095	0.134
Mechanical ventilation time(day)	1.138	0.129	1.062–1.219	<0.001*
Septic shock	1.044	0.043	0.523–2.085	0.903
Protective constraint	3.112	1.135	1.664–5.820	<0.001*
Glucocorticoid	0.916	−0.088	0.421–1.993	0.825
Muscle relaxant	1.391	0.339	0.707–2.736	0.339
Lactic acid	1.346	0.297	1.147–1.581	<0.001*

*, P < 0.05. APACHE Ⅱ, acute physiology and chronic health evaluation Ⅱ.

### 3.3 Development of the scoring scale

**Fig. 3:**
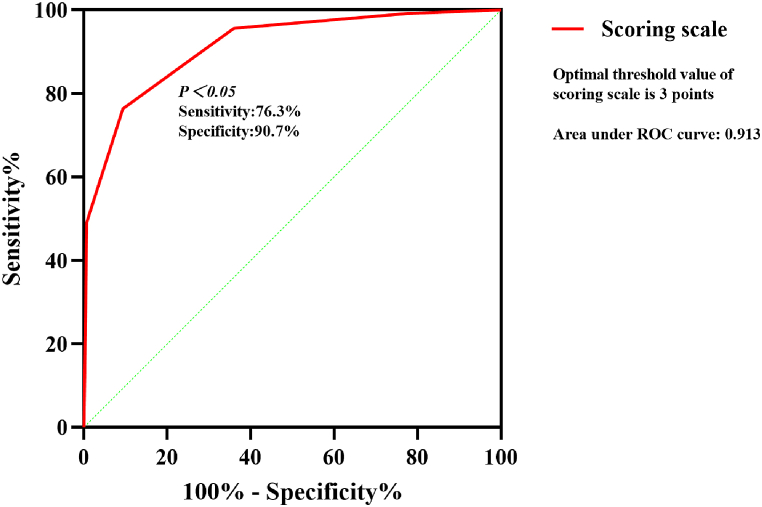
ROC curve of the scoring scale.

Multivariate logistic regression analysis was carried out on the significant findings in univariate analysis and showed 4 clinical characteristics namely APACHE Ⅱ score, mechanical ventilation time, protective constraint and lactic acid were significant predictors of ICU-AW's occurrence in sepsis patients (Table 4). According to the *P* value, all predictors were assigned 1 point (P < 0.001) (Table 5). ROC curve showed that the diagnostic threshold score of the scoring scale was 3 points (sensitivity: 76.3 %, specificity: 90.7 %) (Fig. 3).

**Table 5:** Scoring system for occurrence of ICU-AW in sepsis patients.

Scoring item	Score
Protective constraint	
Yes	1
No	0
APACHE Ⅱ	
≥24	1
<24	0
Mechanical ventilation time	
≥5	1
<5	0
Lactic acid	
≥2.12	1
<2.12	0

APACHE Ⅱ, acute physiology and chronic health evaluation Ⅱ.

## 4 Discussion

Several independent risk factors for ICU-AW have been identified, including a higher severity of illness score, inflammation, multiple organ failure, a longer duration of ICU stay and mechanical ventilation [10,16,17]. Moreover, there is some consensus that sepsis increases the risk of ICU-AW. Research has shown that the pathogenesis of ICU-AW in septic patients may be related to the disruption, impairment, and generation of reactive oxygen species in mitochondrial and ultrastructural components of muscle cells, leading to depletion of cellular adenosine triphosphate (ATP) [18]. Additionally, the excessive degradation of proteins mediated by the ubiquitin-proteasome and autophagy-lysosome systems further exacerbates muscle atrophy and contractile dysfunction [19]. There is evidence suggesting that the systemic cytokine response leads to the local amplification of proinflammatory cytokines within the muscle in sepsis, which may potentially exacerbate the already reduced protein synthesis in skeletal muscle [20]. Animal models of sepsis clearly confirmed that sepsis reduced protein synthesis in skeletal muscle and preferentially inhibited myofibrils and sarcoplasmin synthesis [18]. Sepsis is usually accompanied by acute or chronic organ dysfunction, malignancy, prolonged bedridden treatment, sedative use and glucocorticoid therapy [18]. Therefore, based on the existing literature and our previous studies, we believe that multiple risk factors may interact with each other and ultimately lead to the occurrence of ICU-AW. ICU-AW occurred in 114 of the 264 patients in this study, with an incidence of 43.2 %, indicating that ICU-AW is at a high level in sepsis patients. In the present study, logistic regression analysis revealed that APACHE Ⅱ score, mechanical ventilation time, protective constraint, and lactic acid were risk factors for ICU-AW in patients with sepsis.

### 4.1 APACHE Ⅱ

APACHE is widely used to evaluate the outcomes and characterize the disease severity of ICU patients [21]. The severity of the disease can be quantitatively assessed by scoring. The higher the APACHE Ⅱ score is, the worse the prognosis, the more serious the disease and the higher the risk of death. The APACHE Ⅱ score, reflecting the severity of illness, was an independent risk factor for ICU-AW, which has been demonstrated by several studies [22,23]. The results of this study indicated that the APACHE Ⅱ score of the ICU-AW group was much higher than that of the non-ICU-AW group, and patients with higher APACHE Ⅱ scores were more likely to develop ICU-acquired weakness. Sepsis patients with APACHE Ⅱ scores of 24 points within 24 h of admission should be warned of the occurrence of ICU-AW. An increase of 1 point was associated with a 1.116-fold increase in the incidence of ICU-AW.

### 4.2 Mechanical ventilation time

While in the ICU, between 25 % and 75 % of critically ill patients on mechanical ventilation experience significant skeletal muscle atrophy and weakness [15]. The substantial reduction in muscle mass in the ICU can be linked to alterations in the cross-sectional area of muscle fibers [24]. Porcine models have the capacity to withstand mechanical ventilation for extended periods, allowing for the stimulation of ICU-AW caused by different factors [25]. Pigs that are ventilated, immobilized, and heavily sedated, under the influence of corticosteroids, neuromuscular blocking agents, and/or sepsis, have been utilized to simulate ICU-AW [26,27]. During a period of 5 days, piglets exposed to mechanical ventilation showed minimal alteration in muscle fiber cross-sectional area or specific strength in limb muscles [26,28,29]. However, a reduction in specific force was noted with the co-administration of ventilation and sepsis, corticosteroids, or a combination of all three (steroids, sepsis, and neuromuscular blocking agents) [26,27,29,30]. It was observed that limb and respiratory muscles experienced a significant 45 % reduction in their specific force when using the porcine model [30]. Severe ventilator-induced diaphragmatic dysfunction was speculated to be linked to sarcoplasmic reticulum ryanodine receptor remodeling and abnormal sarcoplasmic reticulum Ca^2+^ leakage at rest, as observed in mechanically ventilated animals, particularly in contrast to hindlimb muscles [31]. Our study showed that a long duration of mechanical ventilation is closely related to the rate of ICU-AW in septic patients. Ballve et al. found that ICU-AW patients in surgery ICUs were older and had longer ICU stays and mechanical ventilation durations [32]. Weakness occurred in 26–65 % of patients who had mechanical ventilation for 5–7 days, and in patients with long-term ventilation (≥10 days), the ICU-AW diagnosis rate was as high as 67 % [33]. The incidence was higher in patients suffering from severe disease [33]. A prospective cohort study of 26 patients with exacerbation of COPD found that the occurrence of ICU-AW was associated with prolonged mechanical ventilation and length of hospital stay [34]. It has been reported that the degree of diaphragm injury after mechanical ventilation for 48 h is positively correlated with the level of ventilator support, leading to diaphragm weakness and even atrophy [35]. Moreover, mechanical ventilation can reduce diaphragm function logarithmically, resulting in abnormal diaphragm function [36]. Most ICU patients require mechanical ventilation to assist with ventilation treatment. Ventilator-induced diaphragmatic dyskinesia is associated with the duration of mechanical ventilation, which may be related to the occurrence of ICU-AW. Prospective cohort studies of the causes of death in ICU-AW patients have shown that an increased duration of mechanical ventilation is associated with increased ICU-AW and mortality [5]. Therefore, it is important to control the duration of mechanical ventilation. However, the mechanism by which prolonged mechanical ventilation causes ICU-AW remains unclear. Patients with prolonged mechanical ventilation often experience protective constraints, increased use of sedatives, use of muscle relaxants, sepsis, hypoxia, and malnutrition, all of which may collectively contribute to the occurrence of ICU-AW [37]. Moreover, although mechanical ventilation is a risk factor, its relationship with ICU-AW may be reciprocal [38]. The ROC curve showed that the diagnostic threshold for the occurrence of ICU-AW in septic patients was 5 days. The longer the duration of mechanical ventilation, the more prone patients are to develop ICU-AW, the prognosis of which is not optimistic and should be considered clinically.

### 4.3 Protective constraint

Patients are immobilized for a long time in the ICU ward. In the traditional model of ICU care for the most severely ill patients, especially during mechanical ventilation, long-term sedation and protective constraints have traditionally been the norm. The protective constraint can cause muscle atrophy, which is reflected in changes in muscle diameter, length and strength [[39], [40], [41]]. Evidence has shown that even in healthy individuals, muscle degeneration can begin as early as 4 h after fixation, with an average daily loss of 1 %–1.3 % of total muscle strength after each day of fixation. In addition, catabolic conditions encountered in critical illness may exacerbate muscle loss in ICU patients [42]. In the disused state, the mass, volume and cross-sectional area of skeletal muscle decreased, and the type of muscle fiber changed from type I to type II [43]. Long-term muscle inactivity can lead to mitochondrial dysfunction, which in turn increases reactive oxygen species (ROS) and ultimately leads to muscle atrophy and dysfunction [43]. Early rehabilitation has been shown to reduce the incidence of ICU-AW, which also supports the effect of protective constraints on ICU-AW development [[44], [45], [46]]. Thus, the protective constraint may be an early identifiable risk factor for targeted interventions.

### 4.4 Lactic acid

Sepsis patients have long-term low oxygen metabolism and insufficient tissue perfusion. Lactic acid is the end product of anaerobic glycolysis of sugars [47]. When the body's cells are hypoxic and blood perfusion is inadequate, anaerobic glycolysis increases, lactic acid is secreted, the hydrogen ion level also increases, and the blood pH value decreases [47]. Acidic environments can stimulate muscle nerve terminal damage and cause Ca^2+^ loss, resulting in decreased muscle nerve excitability, followed by ICU-acquired weakness [48]. 40 % of the body's cell mass is made up of muscle tissue, which physiologically generates 25 % of the body's total lactate (380 mmol/d) [49]. In shock conditions, Daniel et al. showed that in a model of hypokinetic shock, a portion of the lactate comes from the muscles [50]. Hyperlactemia and muscle lactate has also been found to cause muscle weakness in rats [49]. Three different rat models were used to mimic different clinical situations of shock, and muscle lactate were higher in the septic models [49]. The study demonstrated that lactate production during shock states is related, at least in part, to increased Na^+^K^+^-ATPase activity under β2 stimulation [49]. Moreover, a meta-analysis including many studies also suggested that high lactic acid level was a risk factor for ICU-AW [38]. In this study, the serum lactic acid level in the ICU-AW group was higher than that in the non-ICU-AW group (P < 0.001). Multivariate analysis suggested that the risk of developing ICU-AW increased 1.346 times (OR = 1.346) with each 1 mmol/L increase in blood lactic acid level. Blood lactic acid levels were positively correlated with the risk of developing ICU-AW. According to the ROC curve threshold, ICU-AW should be considered when the lactic acid value is greater than 2.12 mmol/L.

### 4.5 Scoring scale

The proposal of the simple scoring scale is helpful for clinicians to predict whether ICU-AW will occur in patients with sepsis and provide support for whether appropriate prevention measures can be taken to prevent the occurrence of ICU-AW. The specificity of the scoring scale in the derivation set was 90.7 %, which means the sepsis patients in the high-risk group authentically have a high incidence of suffering ICU-AW. The high specificity of the scoring scale brings us new questions like whether patients in the high-risk group can be treated in advance to reduce the occurrence of ICU-AW, which is the further research direction of our group. Our score scale also has an unsatisfactory aspect. Different timing of scoring may result in different scores for the same patient. Therefore, it is not possible to prospectively include some patients with sepsis and use their clinical data as a validation set to verify the accuracy of the scoring scale.

## 5 Conclusion

This study identified four risk factors for ICU-acquired weakness in sepsis patients. Clinicians can decrease the occurrence of ICU-AW in sepsis patients by restricting the application of protective constraints, controlling mechanical ventilation time and reducing lactic acid levels during hospitalization. Patients with high APACHE Ⅱ scores at admission should be given more attention. This study was a single-centre study, which may lead to the possibility of false negatives for some factors. Future multicenter randomized controlled trials are needed to explore the risk factors.

## 6 Study limitations

There are some limitations in our study. First, some risk factors may not included as logistic regression analysis heavily relies on the selection of predictor variables. Second, a larger sample size would increase the statistical power available and hence the ability to detect smaller effect sizes. Future studies addressing these limitations will be required to confirm these results.

## Ethical statement

The authors are accountable for all aspects of the work in ensuring that questions related to the accuracy or integrity of any part of the work are appropriately investigated and resolved. This study was conducted in accordance with the Declaration of Helsinki (as revised in 2013) and was approved by the Institutional Ethics Board of the General Hospital of Western Theater Command PLA, (2022EC2-ky035). All participants provided written informed consent to participate in this study.

## Funding statement

This study was supported by the Sichuan Provincial Cadre 10.13039/100005622Health Research Project (2022-1303) and Military Medical Research Project of the General Hospital of Western Theater Command 10.13039/501100017675PLA, Funding (2019LH05).

## Patients’ consent

Informed consent was taken from the participant or legal guardians.

## Data availability statement

Data will be made available on request.

## CRediT authorship contribution statement

**Jiajiao Liu:** Writing – review & editing, Writing – original draft, Validation, Supervision, Software, Methodology, Data curation. **Zhaoxia Xu:** Visualization, Validation, Software, Resources, Investigation, Formal analysis, Data curation, Conceptualization. **Shuhong Luo:** Project administration, Methodology, Investigation, Data curation, Conceptualization. **Yujie Bai:** Visualization, Supervision, Resources, Project administration, Methodology, Formal analysis. **Jian Feng:** Writing – review & editing, Writing – original draft, Supervision, Software, Formal analysis, Conceptualization. **Fuxiang Li:** Writing – review & editing, Writing – original draft, Supervision, Methodology, Investigation, Data curation.

## Declaration of competing interest

Fuxiang Li reports financial support was provided by Sichuan Provincial Cadre 10.13039/100005622Health Research Project. Fuxiang Li reports financial support was provided by Military Medical Research Project of the General Hospital of Western Theater Command PLA Funding. If there are other authors, they declare that they have no known competing financial interests or personal relationships that could have appeared to influence the work reported in this paper.

## List of abbreviations

ICU: intensive care unit
ICU-AW: intensive care unit acquired weakness
APACHE Ⅱ: acute physiology and chronic health evaluation Ⅱ
AUCs: areas under the receiver operating characteristic
MRC-SS: Medical Research Council sum score
MODS: multiple organ dysfunction syndrome
CRRT: continuous renal replacement therapy
PCT: procalcitonin
BMI: body mass index
DIC: disseminated intravascular coagulation
BNP: brain natriuretic peptide
OR: odds ratio
